# Information presentation: enhancing consumer purchase intention through nutritional supplement advertisement design

**DOI:** 10.3389/fnut.2025.1602346

**Published:** 2025-07-11

**Authors:** Shaobo Sun, Jun Cao, Yunyun Wei, Dajun Yang, Ke Guo

**Affiliations:** ^1^Institute of Tourism Research, Huainan Normal University, Huainan, China; ^2^Key Laboratory of Digital-Intelligent Disease Surveillance and Health Governance, North Sichuan Medical College, Nanchong, China

**Keywords:** nutritional supplement advertisement design, purchase intention, structural presentation, consumer psychological mechanisms, technological anxiety

## Abstract

In recent years, how nutritional supplement advertisement design effectively influences purchase intention has emerged as a critical research focus. Traditional studies predominantly emphasize product attributes but overlook how the presentation of product features impacts consumer psychology. Through three experiments, this research investigates the differential effects of internal structural presentation (deconstructed) vs. holistic presentation in nutritional supplement advertisements and elucidates their underlying mechanisms. Experiment 1 (*N* = 305) revealed that the structural presentation group significantly enhanced consumers’ quality perception compared to the holistic presentation group. Experiment 2 (*N* = 229), utilizing a chain mediation model, demonstrated that uncertainty avoidance and panic buying mediate the impact of structural presentation on purchase intention. Experiment 3 (*N* = 304) further identified an interaction between technological anxiety and advertisement visual design: structural presentation exerted stronger effects when high-quality visual design alleviated technological anxiety. This study advances advertisement design theory by highlighting how structural presentation shapes decision-making through psychological mechanisms, offering novel insights for consumer behavior research. The findings provide actionable recommendations for nutritional supplement companies to optimize advertisement design by integrating technical details with visual appeal and strategically addressing consumer anxiety to enhance marketing effectiveness.

## 1 Introduction

The design of nutritional supplement advertisements plays a pivotal role in market promotion, as they not only convey product information (1) but also aim to resonate emotionally with consumers. These advertisements are typically crafted to be both informative and emotionally compelling, seeking to help consumers escape daily information overload while presenting aspirational visions of health and vitality. When discussing nutritional supplement advertisements, primary attention often focuses on their ability to visually demonstrate product nutritional components and efficacy, equating positive advertising messages with actual product benefits (2, 3). However, exceptional nutritional supplement advertisement design extends beyond mere product structure presentation. It can effectively engage consumers through various indirect scenarios unrelated to direct product showcasing. For instance, within healthy lifestyle contexts (4) such as outdoor yoga, morning jogging, or family dining scenarios, advertisements stimulate purchase desire by demonstrating users’ positive transformations after consuming nutritional supplements. Furthermore, advertisements can enhance consumer recognition and trust through authentic user success narratives or by cultivating an atmosphere of wholesome, proactive living (5).

In the expansive field of marketing, the influence of nutritional supplement advertisement design on consumer purchase intention constitutes a subject worthy of in-depth exploration (6). Recent years have witnessed growing research on how nutritional supplement advertisements can more effectively attract consumer purchase intentions, paralleling heightened health consciousness among consumers (7). Studies indicate that specific elements in nutritional supplement advertisements can activate consumers’ health need recognition, thereby influencing purchasing decisions (8). For instance, advertisements emphasizing detailed product composition structures and efficacy appeal to detail-oriented consumers, while those showcasing holistic product outcomes (9) resonate more strongly with effectiveness-driven consumers (10). Previous research predominantly examined the impact of nutritional supplement advertisement design on consumer purchasing behavior from product attribute perspectives. However, as market environments evolve and consumer demands diversify, exclusive focus on product attributes proves insufficient for comprehensively understanding purchase intentions (11). Current studies on nutritional supplement advertisement design frequently remain confined to analyzing advertising elements and product characteristics, while lacking deeper insights into consumer psychology (12) and underlying needs. This limitation results in superficial examinations of advertising’s influence on consumer decision-making and behavior, failing to uncover intrinsic mechanisms. Consequently, investigating how structural presentation and holistic representation in nutritional supplement advertisements synergistically affect consumer purchase intentions (13) represents a highly valuable research domain in marketing. Such exploration not only enhances advertisement design effectiveness but also better aligns with consumer needs and expectations.

A growing body of research suggests that nutritional supplement advertisement design strategies may extend far beyond simple product presentation (14). For instance, through in-depth analysis of consumer preferences, studies reveal that elements in nutritional supplement advertisements can be diversified to include health philosophies, scientific endorsements, and user testimonials, collectively constituting a comprehensive advertising experience rather than being confined to product displays. Similarly, another study demonstrates that nutritional supplement advertisements can integrate natural elements, vibrant colors, and uplifting lifestyle scenarios to evoke consumer resonance and aspiration, thereby enhancing advertisement appeal (15). Building on these findings, this study proposes a conceptual framework for presentation structures in nutritional supplement advertisement design, positing that structural product presentations embody a holistic experience capable of conveying health philosophies, eliciting emotional resonance, and actively guiding consumption behaviors. This broadened conceptualization of presentation structures emphasizes creativity, emotional resonance, and guidance (16). Creativity manifests through unique and novel advertisement content; emotional resonance strengthens persuasive power by establishing emotional connections with consumers (17); guidance reflects the proactive shaping of consumer health perceptions and purchasing behaviors (18). To validate this framework, we propose experimental comparisons of varied advertisement design strategies—structured product presentations versus holistic representations—to assess their differential impacts on purchase intentions (19). Specifically, grounded in cognitive consistency theory, we posit that when nutritional supplement advertisement designs (holistic vs. structural presentations) align with consumers’ cognitive schemas, they broaden attentional scope and enhance information acceptance and identification (20). Consequently, advertisement effects on purchase intentions may be mediated through risk avoidance and panic buying mitigation. When advertisements successfully trigger emotional resonance and align with cognitive patterns, purchase intentions intensify significantly (21). Additionally, consumer technical concerns regarding supplements and visual design feedback may exert moderating effects. For instance, heightened anxiety about manufacturing technologies enhances the appeal of structural product presentations, while visually oriented consumers exhibit stronger preferences for creative and personalized designs.

This study contributes to current debates on nutritional supplement advertisement design and purchase intentions, particularly regarding structural versus holistic presentation paradigms, while engaging consumer engagement and satisfaction research agendas (22). We advance advertisement design theory by proposing an integrated framework balancing structural presentation and holistic visual appeal, demonstrating its correlation with purchase intentions. We educate consumers about nutritional supplements’ health-promoting value (23), emphasizing benefits of aligning choices with long-term wellness goals, and explaining how structurally-holistic advertisement designs amplify purchase intentions. Consequently, this study contributes a strategic framework that enables supplement companies and marketers to design communications where structural information and visual appeal are effectively integrated, ultimately fostering greater consumer purchase intention.

## 2 Theoretical background and research hypotheses

### 2.1 Signaling theory

In many markets, a fundamental condition of information asymmetry exists, where one party in a transaction possesses more or better information than the other. To address the challenges posed by this asymmetry, Signaling Theory provides a robust theoretical framework. First proposed by Spence et al. (24) in the context of the job market, the theory posits that the party with private information (the “sender”) can choose to send observable cues, or “signals,” to the less-informed party (the “receiver”) to credibly convey unobservable qualities. The receiver then interprets these signals to make more informed decisions, thereby reducing their perceived risk and uncertainty (25).

The efficacy of a signal hinges on its credibility, which is primarily determined by its cost. For a signal to be effective, it must be prohibitively costly or counterproductive for a low-quality sender to mimic. This principle, known as the “separating equilibrium,” ensures that only high-quality senders have the incentive to send the signal, making it a trustworthy indicator of underlying quality (26). For example, offering an extensive product warranty is a credible signal of high product reliability because it would be financially ruinous for a company producing unreliable goods to offer the same guarantee. The signal’s cost ensures its honesty.

In the context of the nutritional supplement market, Signaling Theory provides a powerful lens through which to understand consumer decision-making. This market is rife with information asymmetry; consumers often lack the expertise to independently verify a product’s true efficacy, ingredient purity, or manufacturing standards (the unobservable qualities). They are therefore highly motivated to search for credible signals that can help them differentiate high-quality products from low-quality alternatives.

We conceptualize structural presentation as a deliberate and potent signal sent by the firm (the sender) to the consumer (the receiver). By visually deconstructing the product to showcase its internal architecture, scientific formulation, and premium components, the firm signals its confidence in its superior, unobservable attributes. This signal is inherently credible because it is costly and risky for competitors with inferior products to imitate. A firm with substandard ingredients or a simplistic formulation would be unwilling to adopt such transparency, as it would expose their product’s weaknesses. In contrast, a high-quality firm has every incentive to reveal its superior internal architecture to justify a premium price and build consumer trust.

Therefore, grounded in Signaling Theory, we posit that the act of sending this transparent signal directly mitigates the consumer’s perceived uncertainty and enhances their perception of the product’s value. This leads to a more favorable evaluation and a stronger intention to purchase, compared to a holistic presentation which offers no such credible signal and leaves the product’s internal qualities as a “black box.” This theoretical foundation directly underpins our primary hypothesis regarding the superior effectiveness of structural presentation.

### 2.2 How does structural vs. holistic product presentation influence consumer purchase intentions?

In contemporary markets, product design and presentation have become critical for enhancing valuation, particularly in high-tech and luxury sectors (27). In this section, we test the hypothesis that contrasts two distinct presentation styles (28). The first, structural presentation, deconstructs the product to showcase its technical components, scientific processes, and data-driven evidence (29). In direct contrast, holistic presentation emphasizes the integrated, overall benefit and emotional outcome for the user. It avoids technical jargon and instead seeks to convey a complete experience or feeling—such as wellness, energy, or peace of mind—thereby targeting the consumer’s affective processing rather than their analytical cognition (30). We focus on structural presentation, which visually demonstrates a product’s internal architecture, in contrast to holistic presentation, which showcases only the external form. While prior research has acknowledged structural presentation’s role, it has often been confined to esthetics or specific high-tech sectors (31), neglecting its potential in high-uncertainty consumer contexts. Our core argument is that in a market like nutritional supplements, structural presentation transcends mere esthetics; it functions as a powerful signal of quality, transparency, and craftsmanship (32). By revealing the “inside story,” it directly addresses the consumer’s inherent uncertainty, building a foundation of trust and perceived value that a purely holistic presentation cannot (33). This approach transforms the product from a “black box” into a transparent solution, thereby strengthening consumer confidence and their inclination to purchase (34). This study aims to empirically establish this fundamental effect within the nutritional supplement domain, proposing that a strategy of transparency is superior to one of opacity. Therefore, we hypothesize:

*H1*: Structural presentation (vs. holistic presentation) in nutritional supplement advertisement design more effectively enhances consumer purchase intentions.

### 2.3 The mediating mechanism: uncertainty avoidance and panic buying propensity

In high-uncertainty consumer markets, such as nutritional supplements, psychological factors play a pivotal role in shaping purchase behavior (35). Two such factors are central to this study: uncertainty avoidance and technology anxiety. Uncertainty avoidance describes an individual’s discomfort with ambiguous or unknown situations, prompting them to seek credible information or adopt risk-averse strategies to resolve psychological tension. In the context of supplements, where consumers often lack expert knowledge about ingredients and efficacy, this drive to mitigate uncertainty becomes a powerful motivator (36). Simultaneously, in an era of digital marketing, technology anxiety—an individual’s apprehension toward complex technology—critically influences their reception of advertising (37). A visually sophisticated advertisement, such as one featuring detailed internal structural renderings, can be perceived as technologically complex, potentially triggering distrust or disengagement in anxious consumers. Understanding how a specific advertising style—structural presentation—interacts with these pre-existing psychological tensions is therefore essential to unpacking its true effect on consumer intentions.

To explain why structural presentation influences purchase intentions, we propose a serial mediation model involving two key psychological states (38). The first mediator is uncertainty avoidance, which refers to the psychological discomfort and anxiety consumers experience when faced with ambiguity or a lack of clear information about a product (39). We argue that the transparent and detailed nature of structural presentation directly counters this. By providing verifiable information on a product’s composition and mechanisms, it alleviates consumer ambiguity, thus reducing uncertainty avoidance and fulfilling their need for cognitive closure (40). However, in a health-related context, resolving uncertainty does more than just create calm; it validates the product’s importance and can trigger a more urgent emotional response. This leads to our second mediator, panic buying propensity. We define this as a consumer’s tendency toward rapid purchasing driven by a perceived urgent need to secure a benefit or avoid a potential health-related loss (41). When consumers’ uncertainty is resolved and they become convinced of a product’s value, their underlying health anxieties can fuel a powerful desire to act immediately, thereby increasing their panic buying propensity (42). This proposed serial pathway (Structural Presentation → Reduced Uncertainty Avoidance → Increased Panic Buying Propensity → Purchase Intentions) thus reveals a complete psychological chain, explaining how a cognitive input (visual transparency) translates through affective states into a final purchase decision. Accordingly, we hypothesize:

*H2*: Uncertainty avoidance and panic buying propensity sequentially mediate the relationship between structural presentation (internal vs. holistic) in nutritional supplement advertisement design and consumer purchase intentions.

### 2.4 The moderating conditions: technological anxiety and visual design

Building on this core mechanism, we explore the boundary conditions that determine when this effect is amplified or weakened (43). We propose a three-way interaction involving the consumer’s technological anxiety and the advertisement’s visual design. Technological anxiety, or a consumer’s fear of complex technology, is a critical individual trait in modern advertising. For consumers with high technological anxiety, a detailed structural presentation, if poorly executed, could be perceived as intimidating or confusing, thus backfiring and diminishing trust. This is where advertisement visual design becomes a crucial managerial lever (44). A high-quality visual design—characterized by clarity, esthetic appeal, and intuitive layout—can effectively mitigate technological anxiety by making complex information seem accessible, professional, and trustworthy (45). Conversely, a low-quality design will exacerbate this anxiety, rendering the structural information ineffective (46). We therefore posit a complex interplay: the positive effect of structural presentation is strongest when consumers have low technological anxiety, or when their high technological anxiety is successfully counteracted by a high-quality, reassuring visual design (47). This three-way interaction provides a highly nuanced understanding of how a consumer’s intrinsic traits and a firm’s executional tactics jointly shape advertising effectiveness, moving beyond simplistic main effects to offer a more realistic framework for marketers. Based on this, we hypothesize:

*H3*: Technological anxiety and advertisement visual design interactively moderate the relationship between structural presentation (internal vs. holistic) in nutritional supplement advertisement design and consumer purchase intentions.

While prior research confirms the importance of product presentation, its role in high-uncertainty contexts remains underexplored. Existing studies on structural presentation have largely focused on esthetics or its effects in sectors like electronics, neglecting its underlying psychological mechanisms and boundary conditions. This study addresses these gaps through four key theoretical contributions (48). First, we introduce a novel perspective by shifting focus from traditional ad frames to “structural presentation” (internal vs. holistic) within the unique context of nutritional supplements, a domain where consumer trust and perceived efficacy are critical. This approach moves beyond esthetics to examine how structural details influence high-stakes consumer decisions (49). Second, to deepen the mechanistic understanding, we propose and validate a pioneering serial mediation model. This model, incorporating “uncertainty avoidance” and “panic buying propensity,” aims to uncover the deep psychological pathway—the “how”—that connects structural presentation to purchase intention, offering a more nuanced explanation than a simple main effect (50). Third, for contextual expansion, we introduce “technological anxiety” as a novel moderator. This allows us to investigate the complex boundary conditions of the digital era, exploring “when” the impact of structural presentation is altered by the interplay of consumer traits and ad design. Finally, through theoretical integration, our study constructs a sophisticated model featuring both serial mediation and a three-way interaction (51). This integrated framework not only extends the boundaries of advertising psychology but also provides actionable insights for marketing in uncertain environments. The subsequent sections will detail the hypotheses that constitute this model.

**Figure 1:**
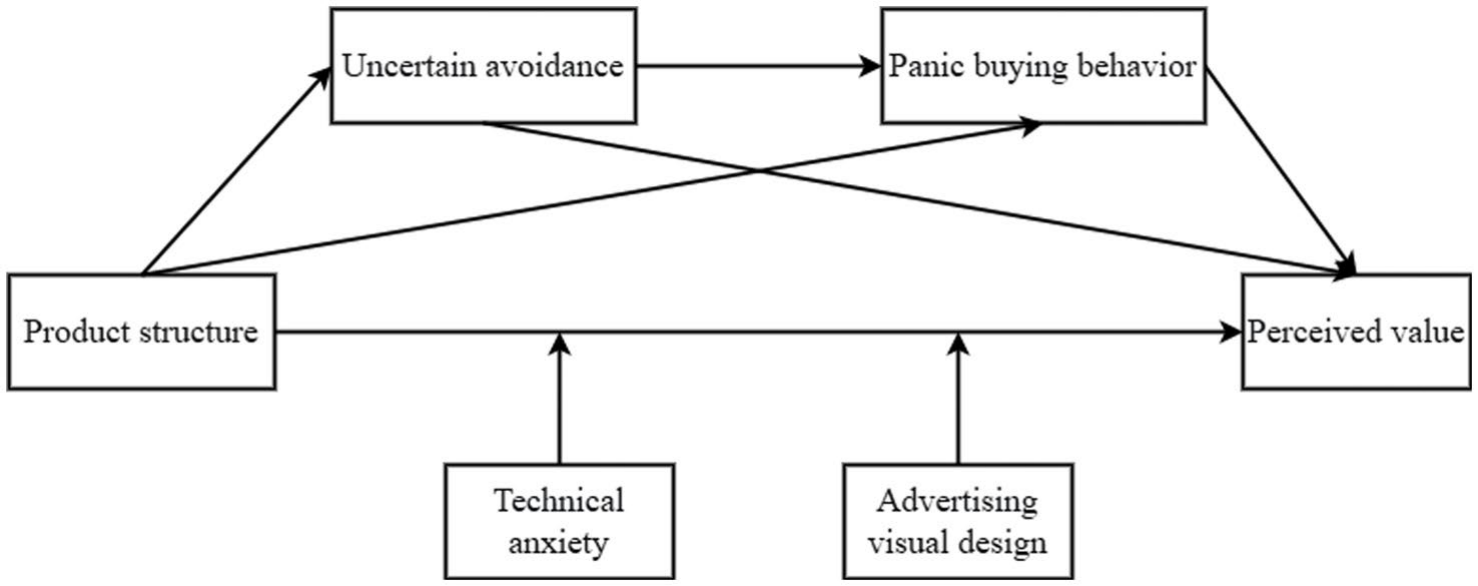
Theoretical model of this study.

The theoretical model for this study is shown in Figure 1.

## 3 Research design

To provide a comprehensive and rigorous test of our theoretical model, we adopted a programmatic, three-study experimental approach (52). This sequential design methodology is considered a robust strategy for building and testing causal theories in a cumulative manner, moving systematically from demonstrating a core effect to explaining its underlying process and identifying its boundary conditions (53, 54). This research employs three sequential experiments to systematically test our hypotheses, each designed to progressively uncover how structural presentation (internal vs. holistic) in nutritional supplement advertising impacts purchase intention. First, Experiment 1 (The “What”) establishes the foundational main effect (H1) by directly comparing internal and holistic presentations, confirming which format more effectively boosts purchase intention and providing a baseline for our model. Building on this, Experiment 2 (The “Why”) investigates the underlying mechanism by testing the proposed serial mediation pathway (H2), aiming to reveal whether the effect is driven by reduced uncertainty avoidance and subsequent panic buying propensity. Finally, Experiment 3 (The “When”) addresses the boundary conditions (H3) by introducing technological anxiety and advertisement visual design as moderators, testing how their three-way interaction alters the main effect. Collectively, this three-study package forms a coherent chain of evidence, moving from the foundational effect to its mechanism and boundaries, thereby offering a robust, multi-faceted validation of our theoretical model with significant theoretical and practical implications. Collectively, this three-study package provides convergent evidence for our model, enhancing confidence in the findings and balancing internal with external validity (55, 56). By progressing from effect to mechanism and then to boundaries, our research offers a robust, multi-faceted validation of our theory (57). The demographic characteristics of the participants in each of the three experiments are detailed in their respective methods sections (58) (Table 1).

**Table 1:** Demographic information.

Variable	Items	Experiment 1 (*N* = 305)		Experiment 2 (*N* = 229)		Experiment 3 (*N* = 304)	
		Frequency	Proportion	Frequency	Proportion	Frequency	Proportion
Gender	Male	150	49.2%	110	48%	152	50%
	Female	155	50.8%	119	52%	152	50%
Age	18–25 years old	102	33.4%	81	35.4%	100	32.9%
	26–40 years old	99	32.5%	84	36.7%	103	33.9%
	41–60 years old	51	16.7%	30	13.1%	51	16.8%
	Over 61 years old	53	17.4%	34	14.8%	50	16.4%
Education background	Primary school	12	3.9%	6	2.6%	13	4.3%
	Junior high school	13	4.3%	5	2.2%	11	3.6%
	Technical secondary school, High school	13	4.3%	6	2.6%	10	3.3%
	Junior college	12	3.9%	6	2.6%	12	3.9%
	Undergraduate college	213	69.8%	183	79.9%	216	71.1%
	Postgraduate	22	7.2%	10	4.4%	21	6.9%
	Doctor-postgraduate	20	6.6%	13	5.7%	21	6.9%

Participants were recruited online through randomly distributed surveys. The required sample size for each experiment was determined using an *a priori* power analysis conducted in G*Power (Version 3.1). The analysis was designed to detect a medium effect size (Cohen’s d = 0.5) with a high statistical power of 0.90 (1 − β) at a stringent alpha level of 0.01. The results indicated that a minimum of 120 participants per experiment was necessary. To enhance the reliability of our findings and account for potential sample attrition (e.g., from non-responses or incomplete data), we adopted a conservative approach by oversampling. Consequently, we collected data from over 200 participants for each of the three experiments. The demographic characteristics of the participants in the three experiments. For the measurement scales of this study, please refer to Table 2.

**Table 2:** Variable scale.

Variable name	Item	Reference source
Independent variable		Kang et al. (92)
Uncertainty avoidance	I prefer to purchase nutritional supplements from familiar retailers over unfamiliar ones, because I am more confident in the quality and origin of their products	Wang et al. (61)
	When purchasing nutritional supplements, I favor familiar stores and merchants over unfamiliar environments, as they provide greater assurance regarding product quality and sourcing	
	I prefer shopping environments with clear rules and procedures; when purchasing nutritional supplements, I favor predictable experiences over those that are spontaneous or unpredictable.	
Panic buying behavior	The fear of not having nutritional supplements when I need them causes me to buy more than I immediately need.	Dash et al. (62)
	The thought of essential nutritional supplements running out of stock makes me feel panicked, which is why I prefer to buy in bulk.	
	One way I alleviate feelings of uncertainty is by making sure I have a large supply of the nutritional supplements I need at home.	
	Panic drives me to buy more nutritional supplements to stock up at home.	
	My fear of scarcity has led me to buy nutritional supplements that are outside of my usual choices.	
Technology anxiety	I feel apprehensive about using technology-enhanced nutritional supplements.	Meuter et al. (63)
	The technical terms associated with technology-based nutritional supplements sound like confusing jargon to me.	
	I avoid technology-based nutritional supplements because I am unfamiliar with these new technologies.	
	I hesitate to use most forms of technology-based supplements because I worry that making an improper choice or using them incorrectly could lead to an uncorrectable mistake affecting my health.	
Advertisement visual design	Overall, the visual elements of this nutritional supplement advertisement (e.g., color, imagery, lighting, size, shape) are of high quality	Shaouf et al. (64)
	Overall, the visual design elements used in the advertisement make it appear professional and well-designed.	
	This nutritional supplement advertisement is visually attractive.	
	Overall, the visual elements in this nutritional supplement advertisement are pleasing to the eye.	
Purchase intention	If you were to purchase a nutritional supplement, could you indicate the likelihood of you buying this brand of nutritional supplement?	Toldos-Romero and Orozco-Gómez (65)

## 4 Experiment 1: product deconstruction and consumers’ perceived nutritional quality

### 4.1 Design and procedures

Experiment 1 was designed to examine how product structure enhances consumers’ perception of nutritional quality (Test Hypothesis H1). We conducted a single-factor between-subjects variance experiment (product structure: deconstructed vs. holistic). We recruited 310 participants through the professional questionnaire platform Credamo,^1^ and 5 participants were excluded for failing the attention check. All participants were randomly assigned to either the deconstructed group (153 participants) or the holistic group (152 participants).

**Figure 2:**
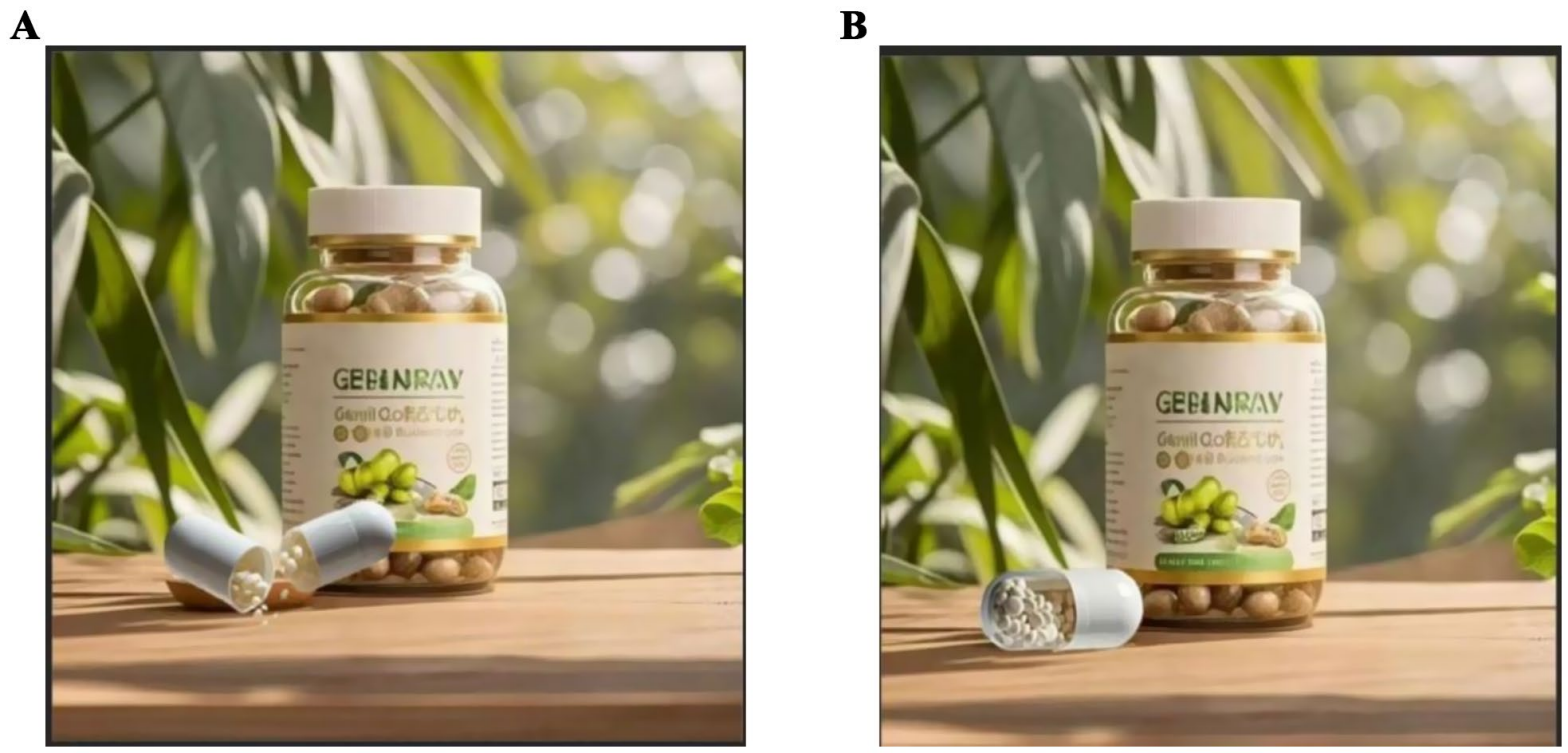
Stimulus figure for Experiment 1.

*Experimental procedure*: First, we asked participants in both groups to imagine they were shopping for nutritional supplements in a mall and came across different advertisements for the same product. Next, the deconstructed group was presented with the following advertising copy for the nutritional supplement: “High-quality nutritional supplement, designed for a healthy life, rich in various vitamins and minerals, helps to make up for daily dietary deficiencies, enhances immunity, and promotes health,” as shown in Figure 2A. In contrast, the holistic group was shown the following advertising copy: “Nutritional supplement for dietary nutrition, promotes health, enhances immunity, and improves specific conditions,” as shown in Figure 2B. Subsequently, participants were asked to answer questions measuring perceived value, such as: “If you were to purchase a nutritional supplement, could you indicate the likelihood of you buying this brand of nutritional supplement?” (59) (1 = strongly disagree, 7 = strongly agree). Finally, we collected demographic information from the participants. Detailed information is presented in Figure 2.

### 4.2 Results and discussion

*Main effect test*: We conducted a one-way ANOVA with product structure as the independent variable and perceived nutritional quality as the dependent variable. The results revealed that participants in the deconstructed group (M = 6.107, SD = 1.131) had a significantly stronger perception of nutritional quality than those in the holistic group (M = 4.373, SD = 0.616), *F*(1, 303) = 275.988, *p* < 0.001, η^2^ = 0.477, thus confirming Hypothesis H1.

*Control variable analysis*: Given that Kumpulainen et al. (60) found that consumers’ gender significantly affects their perception of a product’s nutritional quality, we conducted a one-way ANOVA with gender as the independent variable. The experimental results indicated that gender had no significant impact on consumers’ perception of nutritional quality [*F*(1, 303) = 1.803, *p* = 0.18]. Therefore, gender did not influence the experimental results, further validating Hypothesis H1.

Experiment 1 provides preliminary evidence for our prediction that deconstructing the internal structure of nutritional products enhances consumers’ perception of the product’s quality (H1). In other words, participants under the deconstructed product structure condition demonstrated a stronger perception of the product’s quality compared to those under the holistic product structure condition.

## 5 Experiment 2: chain mediation test of uncertainty avoidance and panic buying behavior

### 5.1 Design and procedures

Experiment 2 designed a single-factor between-subjects experiment (product structure: deconstructed vs. holistic), aiming to examine the chain mediation effect of uncertainty avoidance and panic buying behavior on the relationship between product structure and consumers’ perceived nutritional quality (Test Hypothesis H2). We recruited 230 participants through the professional questionnaire platform Credamo (see text footnote 1), and one participant was excluded for having a strongly consistent questionnaire response. All participants were randomly assigned to either the deconstructed group (122 participants) or the holistic group (107 participants).

**Figure 3:**
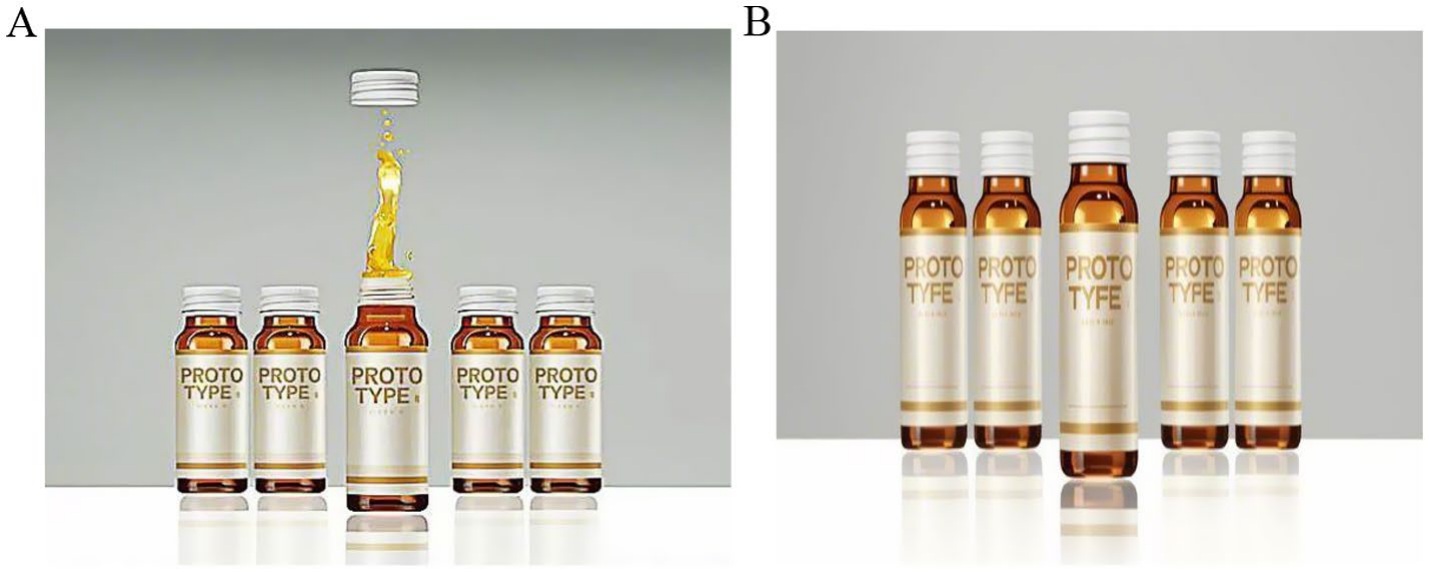
Stimulus materials for Experiment 2.

*Experimental procedure*: First, we asked participants in both groups to imagine that they had been feeling tired lately, and after a doctor’s diagnosis, they were told that their body was lacking in nutrition and were advised to go to the mall to buy nutritional supplements as consumers. Next, participants in both groups would see the product introduction consistent with Experiment 1, but with different nutritional supplement structure images provided, as shown in Figure 3. Immediately after, participants were asked to answer questions measuring uncertainty avoidance, such as: “I prefer to purchase nutritional supplements from familiar retailers over unfamiliar ones, because I am more confident in the quality and origin of their products” (1 = strongly disagree, 7 = strongly agree) (61). Then, we asked participants about their panic buying behavior, such as: “My fear of scarcity has led me to buy nutritional supplements that are outside of my usual choices.” (1 = strongly disagree, 7 = strongly agree) (62). Participants also answered questions measuring perceived value (1 = strongly disagree, 7 = strongly agree) (59). Finally, we collected demographic information from the participants. Detailed information is presented in Table 1.

### 5.2 Results and discussion

*Main effect test*: We conducted a one-way ANOVA with product structure as the independent variable and perceived nutritional value as the dependent variable. The perceived nutritional value of the nutritional supplement was (M_deconstructed = 6.39, SD_deconstructed = 0.816; M_holistic = 4.582, SD_holistic = 0.662), *F*(1, 227) = 332.954, *p* < 0.001, η^2^ = 0.595. This indicates that participants in the deconstructed group had a stronger perception of the nutritional quality of the supplement than those in the holistic group, thus further confirming H1.

**Figure 4:**
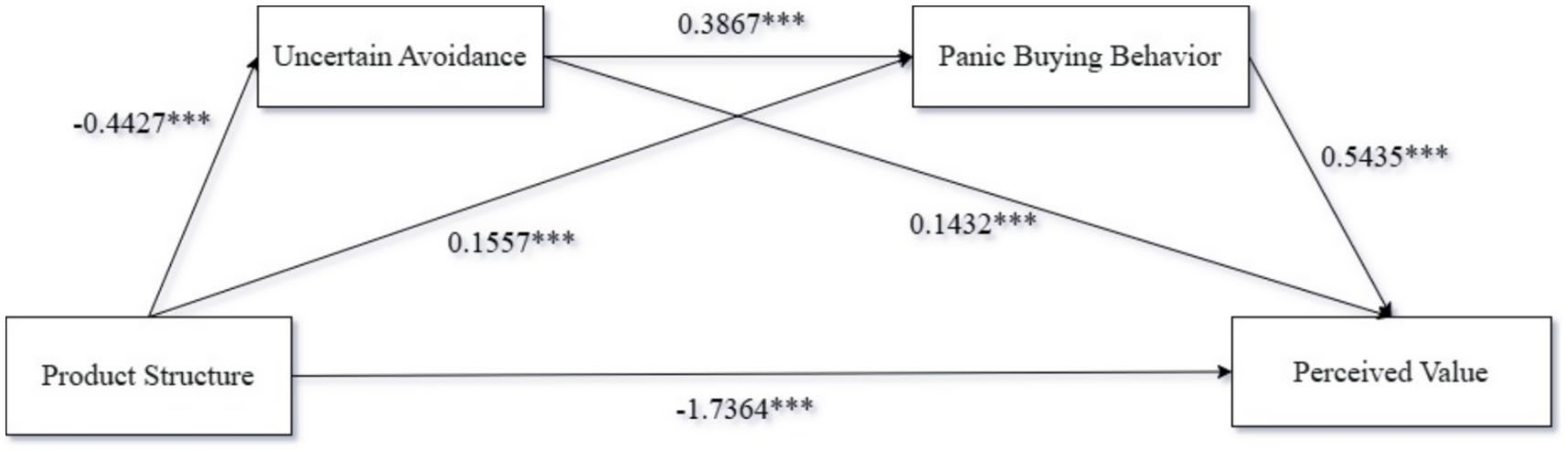
Diagram of Experiment 2 results. * indicates *P* <0.05, ** indicates *P* <0.01, *** indicates *P* <0.001.

*Chain mediation effect test*: We used PROCESS Model 6 to examine the chain mediation effect of uncertainty avoidance and panic buying behavior. Specifically, we took product structure as the independent variable, perceived nutritional value as the dependent variable, and uncertainty avoidance and panic buying behavior as the mediator variables. The analysis showed that product structure had a significant impact on the perception of the nutritional supplement’s quality (β = −1.7364, *p* < 0.001, 95%CI = [−1.9038, −1.569]), product structure had a significant impact on uncertainty avoidance (β = −0.4427, *p* < 0.001, 95%CI = [−0.7101, −0.1753]), product structure had a significant impact on panic buying behavior (β = 0.1557, *p* < 0.001, 95%CI = [0.0438, 0.2677]), and there was a significant effect between uncertainty avoidance and panic buying behavior (β = 0.3867, *p* < 0.001, 95%CI = [0.3332, 0.4403]). Specifically, uncertainty avoidance played a mediating role between product structure and the perception of the nutritional supplement’s quality (β = −0.0634, SE = 0.0355, 95%CI = [−0.1399, −0.0006]); panic buying behavior played a mediating role between product structure and the perception of the nutritional supplement’s quality (β = 0.0846, SE = 0.0400, 95%CI = [0.0197, 0.1737]); uncertainty avoidance and panic buying behavior had a chain mediating effect on the relationship between product structure and the perception of the nutritional supplement’s quality (β = −0.0794, SE = 0.0313, 95%CI = [−0.1488, −0.0257]), as shown in Figure 4. This indicates that the results of Experiment 2 provide evidence for the chain mediating effect of uncertainty avoidance and panic buying behavior, thus confirming Hypothesis H2. Detailed information is presented in Figure 4.

In Experiment 2, we have verified the chain mediation effect of uncertainty avoidance and panic buying behavior on the relationship between product structure and the perception of nutritional supplement quality, thus confirming Hypothesis H2. Specifically, the difference in product structure increases consumers’ uncertainty avoidance regarding product selection, which in turn reduces their anxiety and perception of uncertainty, prompting them to engage in panic buying behavior. However, this behavior further affects consumers’ perception of the quality of nutritional supplements.

## 6 Experiment 3: the dual moderating effect of visual advertising design and technology anxiety

### 6.1 Design and procedures

Study 3 employed a 2 (product structure: deconstructed vs. holistic) × 2 (visual advertising design: high vs. low) × 2 (technology anxiety: high vs. low) between—subjects design, aiming to examine the moderating effects of technology anxiety and visual advertising design on the relationship between product structure and consumers’ perceived nutritional quality (Test Hypothesis H3). We recruited 304 participants through the professional questionnaire platform Credamo (see text footnote 1). All participants were randomly assigned to either the deconstructed group (152 participants) or the holistic group (152 participants).

**Figure 5:**
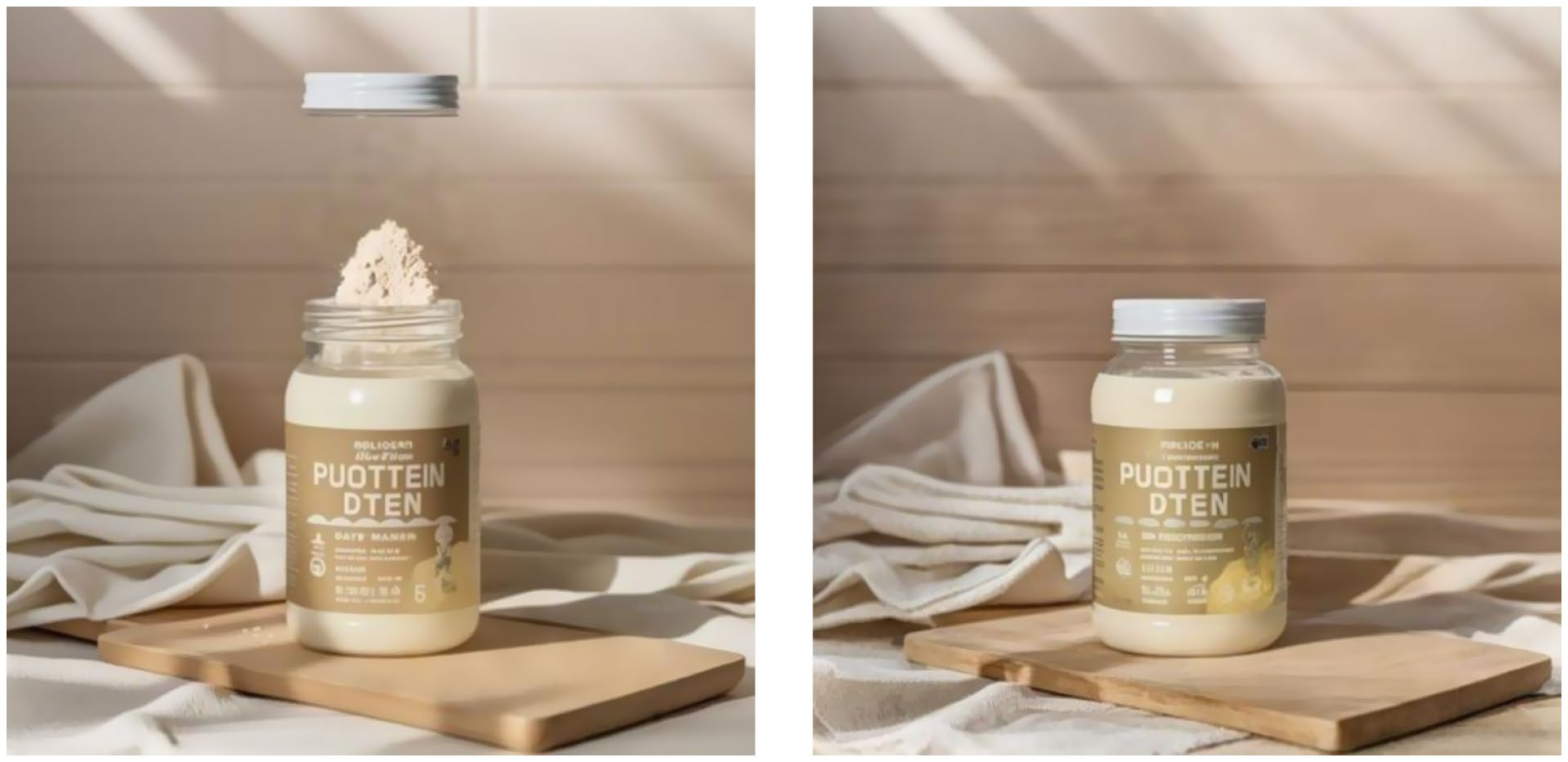
Stimulus figure for Experiment 3.

*Experimental procedure*: First, we asked participants in both groups to imagine that, given the severe situation of the epidemic, multiple experts have suggested that residents prepare nutritional supplements at home. Next, participants in both groups would see the product introduction consistent with Experiment 1, but with different nutritional supplement structure images provided, as shown in Figure 5. Immediately after, we asked participants about their technology anxiety, for example, “I feel apprehensive about using technology-enhanced nutritional supplements.” (1 = strongly disagree, 7 = strongly agree) (63). Then, participants answered questions about the visual advertising design, such as, “Overall, the visual elements of this nutritional supplement advertisement (e.g., color, imagery, lighting, size, shape) are of high quality” (1 = strongly disagree, 7 = strongly agree) (64). Participants also answered questions measuring the perceived value of the nutritional supplement (59). Finally, we collected demographic information from the participants. Detailed information is presented in Table 1.

### 6.2 Results and discussion

*Main effect test*: We conducted a one-way ANOVA with product structure as the independent variable and perceived nutritional quality as the dependent variable. Participants in the deconstructed group (M = 6.07, SD = 1.124) had a stronger perception of nutritional quality than those in the holistic group (M = 4.598, SD = 0.624), *F*(1, 302) = 200.785, *p* < 0.001, η^2^ = 0.399, thus confirming Hypothesis H1.

**Figure 6:**
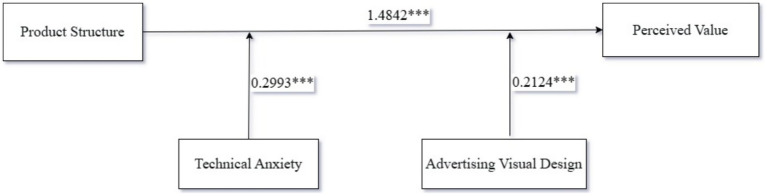
The results of Experiment 3. * indicates *P* <0.05, ** indicates *P* <0.01, *** indicates *P* <0.001.

*Moderating effect analysis*: We took product structure as the independent variable and perceived value as the dependent variable, and examined the dual moderating effect of technology anxiety and advertising visual design using Process Model 2. The experimental results showed that the interaction among product structure, technology anxiety, and advertising visual design had a significant impact on consumers’ perceived value of the nutritional supplement [R^2^ = 0.0642, *F*(2, 298) = 25.9365, *p* < 0.001]; the interaction between product structure and technology anxiety had a significant impact on the perceived value of the nutritional supplement (β = 0.3411, *p* < 0.001, 95%CI = [0.1802, 0.502]); the interaction between product structure and advertising visual design had a significant impact on the perceived value of the nutritional supplement (β = 0.3495, *p* < 0.001, 95%CI = [0.1105, 0.5885]). Specifically, the main effect of product structure on perceived value was significant (β = 1.4842, *p* < 0.001, 95%CI = [1.322, 1.6462]); technology anxiety had a significant impact on perceived value (β = 0.2993, *p* < 0.001, 95%CI = [0.2188, 0.3797]); advertising visual design had a significant impact on perceived value (β = 0.2142, *p* < 0.001, 95%CI = [0.0947, 0.3337]). Therefore, product structure, technology anxiety, and advertising visual design had a significant impact on the perceived nutritional value of the supplement, as shown in Figure 6, thus confirming Hypothesis H3.

In Experiment 3, we have verified that product structure, technology anxiety, and advertising visual design have a significant moderating effect on the perception of the nutritional value of nutritional supplements. Specifically, the deconstructed structure display of product structure can enhance consumers’ confidence in product performance, but this positive effect is moderated by technology anxiety. When the level of technology anxiety is high, advertising visual design can alleviate consumers’ anxiety through emotional and intuitive presentation methods, thereby enhancing the positive effect of product structure on the perception of nutritional value.

## 7 General discussion

This study conducted three experiments to delve into the impact of structural display in nutritional supplement advertising (internal structure display vs. holistic display) on consumers’ purchase intention and verified three core hypotheses. First, this study found that the internal structure display in nutritional supplement advertising is more effective than the holistic display in enhancing consumers’ purchase intention (H1). This finding reveals the advantage of internal structure display in providing detailed product information and transparency, which helps build consumer trust and thus promotes purchase decisions. Second, the study confirmed the chain mediation effect of uncertainty avoidance and panic buying between structural display and purchase intention (H2). When consumers face the choice of nutritional supplements, the internal structure display, by reducing product uncertainty, may trigger panic buying behavior, especially in the current context where health awareness is increasingly growing, and consumers are more inclined to choose products that can intuitively display their internal quality and ingredients. Finally, the study also revealed the interactive effect of technology anxiety and advertising visual design on the effectiveness of structural display (H3). When advertising visual design can effectively alleviate consumers’ technology anxiety, the promotional effect of internal structure display on purchase intention is more significant. This indicates that when designing nutritional supplement advertisements, it is essential to fully consider the target audience’s level of technology anxiety and enhance the acceptance and persuasiveness of advertising information by optimizing visual design.

### 7.1 Theoretical contributions

This study makes four distinct and substantive contributions to the literature on Signaling Theory, consumer psychology, and health communication. Each contribution builds upon the last, moving from a broad theoretical extension to specific mechanistic and contextual refinements. First, we extend the conceptual boundaries of Signaling Theory by identifying and empirically validating visual deconstruction as a novel and potent signaling strategy (65). Foundational research on Signaling Theory has traditionally focused on discrete, often costly, signals such as warranties, brand advertising expenditure, or pricing (66). Our study advances this literature by demonstrating that a message’s very presentation format can function as a powerful, non-obvious signal of unobservable product quality. We conceptualize structural presentation not merely as an esthetic choice but as a strategic act of transparency that is costly for low-quality competitors to mimic (29). By revealing a product’s internal architecture, a firm signals supreme confidence in its underlying attributes—a signal that would expose the inadequacies of inferior products. This contribution advances the work of scholars like (25) by shifting the focus from what is said (e.g., a claim) to how it is shown, providing a new theoretical lens for analyzing visual communication as a strategic tool for resolving information asymmetry (67).

Second, our research contributes a critical finding to the consumer behavior literature by establishing a clear persuasive hierarchy between structural and holistic presentations in high-uncertainty contexts (68). While much advertising research has championed the efficacy of holistic, emotionally resonant appeals (69), our findings challenge this conventional wisdom within the nutritional supplement domain (70). We demonstrate that for products where consumer uncertainty is high and trust is paramount, an informational, transparency-focused approach (structural presentation) is significantly more effective at driving purchase intentions than an affect-based, holistic one (71). This establishes a key principle: when a consumer’s primary psychological barrier is a lack of verifiable information, signals of transparency can override signals of esthetic appeal (72). This provides a crucial evidence-based counterpoint to studies that broadly advocate for esthetic dominance and offers a more nuanced framework for marketers choosing between rational and emotional advertising strategies (73).

Third, we make a significant contribution by illuminating the specific psychological micro-processes through which a visual signal translates into behavioral intention. Signaling Theory adeptly explains that signals work (74), but it often treats the receiver’s interpretive process as a “black box.” Our study opens this box by validating a novel sequential mediation pathway: Structural Presentation → Reduced Uncertainty Avoidance → Increased Panic Buying Propensity → Purchase Intentions. This is a key theoretical advancement for two reasons. (a) It moves beyond a simplistic “signal → trust → purchase” model by identifying a more complex, two-step affective-conative process. (b) It reframes “panic buying propensity” in this context not as an irrational herd behavior, but as a goal-oriented urgency triggered by the successful resolution of uncertainty (75). By uncovering this specific chain reaction, our study provides a more granular and dynamic process model that enriches our understanding of how consumers psychologically digest and act upon quality signals in health-related decisions (76).

Finally, we refine Signaling Theory by uncovering a crucial boundary condition that underscores the principle of signal-receiver-context congruence. We demonstrate that a signal’s effectiveness is not absolute but is contingent upon a complex, three-way interaction between the signal (presentation style), the receiver’s disposition (technological anxiety), and the reception context (advertisement visual design) (77). This moves far beyond identifying simple moderators. Our finding contributes a sophisticated contingency perspective, showing that even a credible signal (78) like structural presentation can fail if the receiver is not psychologically prepared to process it, or if the context in which it is delivered is threatening. We show that a “warm” and empathetic visual design can function as a “meta-signal” that mitigates the receiver’s anxiety, thereby unlocking the power of the primary signal (79). This advances signaling research by highlighting that effective signaling in the digital age requires not only sending a credible signal but also actively managing the reception environment to align with the receiver’s psychological state (38).

### 7.2 Managerial implications

This study offers novel perspectives on enhancing consumer purchase intentions through structural presentation (80). Traditional marketing often prioritizes showcasing holistic esthetics or unique selling propositions to capture attention. However, structural presentation elevates purchase intentions by revealing a product’s internal architecture and artisanal details, fostering deeper consumer understanding (81). Its efficacy stems from addressing demands for transparency and authenticity (82). In markets plagued by information asymmetry, consumers struggle to assess product quality, making structural presentation a critical tool for value evaluation (83). This study demonstrates that marketers can amplify product appeal by optimizing structural presentation—for instance, detailing production processes, ingredient sourcing, and quality control in supplement advertisements to emphasize reliability. Coupled with sophisticated visual design and compelling copywriting, such strategies further stimulate purchase desire. Crucially, effective structural presentation transcends mere internal exposure; it requires strategic design to communicate core values and unique propositions through intuitive, accessible formats (84). Thus, marketers must prioritize professional and creative execution to ensure structural presentation resonates with target audiences and drives positive purchasing behaviors.

The findings hold profound societal implications for the supplement industry and consumers (85). In today’s information-saturated markets, where choice overload intensifies consumer indecision, strategically designed structural presentations not only shape purchasing decisions but also subtly guide public health awareness and consumption norms (86). Supplement retailers can leverage these insights to communicate health priorities through structural presentation designs. Specifically, internal structural presentation reduces product uncertainty by visually verifying quality and composition, thereby boosting purchase intentions. This approach not only enhances sales but also cultivates responsible health consumption mindsets. Furthermore, marketers should exploit the sequential mediation of uncertainty avoidance and panic buying propensity to foster proactive health attitudes via advertising (87). Unlike conventional health education, psychologically informed strategies—such as showcasing premium ingredients and scientific manufacturing within aspirational lifestyle contexts—resonate more deeply, motivating consumers to pursue healthier lifestyles (88). For example, advertisements integrating high-quality internal components with familial warmth or wellness philosophies can create uplifting narratives that captivate attention and inspire health-driven aspirations.

This study also found that the research has important guiding significance for the formulation of marketing strategies in the nutritional supplement industry. In today’s world, where technology is increasingly advanced, the issue of technological anxiety among consumers is becoming more prominent, posing new challenges for the design of nutritional supplement advertisements. Technological anxiety often leaves consumers feeling confused and uneasy when faced with complex advertising information (89), which in turn affects their purchasing decisions. As an important means of conveying product information, the effectiveness of advertising visual design largely depends on how it interacts with technological anxiety. This study found that when advertising visual design can fully consider the level of consumers’ technological anxiety and present the internal structure display in a simple and easy-to-understand way, it can significantly reduce consumers’ technological anxiety and enhance their willingness to purchase (90). In contrast, if the advertising visual design is too complex or difficult to understand, it may exacerbate consumers’ technological anxiety and thus reduce their willingness to purchase. Therefore, for the nutritional supplement industry, a successful marketing strategy should make full use of the interactive effect of advertising visual design and technological anxiety, guiding consumers’ purchasing behavior through reasonable internal structure display and easy-to-understand visual design. This not only helps to enhance the market competitiveness of products (91) but also better meets consumers’ health needs.

Our findings regarding advertising presentation style offer clear and actionable guidance for companies marketing nutritional supplements. The primary implication is a strategic shift from ‘black box’ marketing, which focuses on external packaging and brand imagery, to a more transparent ‘glass box’ approach. Specifically, managers should invest in high-quality visual assets like 3D renderings or animations that vividly showcase the product’s internal architecture—be it its multi-layer technology, scientific formulation, or ingredient purity. This visual strategy should be paired with a two-pronged communication tactic that mirrors our psychological findings: first, leverage this transparency to proactively build trust and resolve consumer uncertainty; second, once trust is established, effectively create a sense of urgency or ‘fear of missing out’ to trigger the purchase. The success of this transparent strategy, however, is contingent on careful execution: visuals must be tailored to the audience’s technological anxiety, and investing in professional, high-quality design is not optional but essential, as a poorly executed graphic will undermine credibility and negate all benefits.

In conclusion, based on our research findings, we propose two distinct and actionable strategic blueprints. For consumers with low technological anxiety, such as the technologically proficient youth, analytical consumers, and early adopters, “structural details” should be interpreted as an in-depth showcase of technological sophistication. This includes complex 3D-rendered animations illustrating multi-layered absorption processes and detailed scientific data visualizations, as this cohort perceives complexity as a credible signal of superior quality and efficacy. Correspondingly, “appealing visual design” for this group should embody an avant-garde, modern, and technologically-inspired esthetic, utilizing futuristic color palettes and fluid animations to cultivate a perception of cutting-edge science. Conversely, for consumers with high technological anxiety, including older demographics, safety-conscious consumers, and late adopters, “structural details” must focus on providing reassurance through simplification and metaphor. Rather than showcasing intimidating complexity, this approach uses clear infographics that translate technical processes into digestible benefits, such as a “shield” icon representing antioxidant protection. The “appealing visual design” for this segment should therefore convey a warm, humanistic, and trustworthy esthetic, achieved through natural colors, soft shapes, and relatable figures like a trusted physician, in order to alleviate anxiety and build trust.

### 7.3 Limitations and further research

#### 7.3.1 Limitations of the current study

First, a primary limitation pertains to the generalizability of our findings. Our research utilized online convenience samples, which, while common in experimental consumer research, may not be fully representative of the broader population of supplement consumers. The demographic and psychographic characteristics of our participants could differ from those of other consumer segments, thus warranting caution when extrapolating our results. Second, our methodology, which relied on controlled experiments with hypothetical purchase scenarios, prioritized internal validity at the expense of external validity. While this approach allowed for a clean test of our hypotheses, it does not fully capture the complexity of real-world purchase decisions, where factors such as brand loyalty, pricing, and in-store promotions interact with advertisement design. Finally, the stimuli created for our experiments were for a single, generic type of nutritional supplement. The observed effects of structural presentation may vary depending on the product sub-category (e.g., basic vitamins vs. complex herbal formulations) or the consumer’s pre-existing familiarity with the product.

#### 7.3.2 Directions for future research

Building upon these limitations, several avenues for future research emerge. First, to address the issue of generalizability, future studies should replicate our findings using more diverse and representative samples. Researchers could explicitly investigate how demographic variables (e.g., age, education level) or psychographic traits (e.g., health consciousness, skepticism toward advertising) moderate the effects we observed, thereby identifying more precise target consumer profiles. Second, future research should aim to build more comprehensive theoretical models that integrate the variables we studied with other key marketing signals. For instance, how does the signal of structural presentation interact with the signal of a high price or a well-established brand name? Investigating these interactions would provide a more holistic understanding of consumer decision-making and enhance the study’s external validity. Finally, while our study identified a key psychological mechanism (uncertainty avoidance → panic buying), future research could delve deeper into other parallel psychological processes. For example, does structural presentation also enhance perceived product efficacy, cognitive fluency, or a sense of consumer empowerment? Uncovering these additional mediators would offer a richer explanation for why transparency is so persuasive and could lead to the development of even more effective marketing strategies.

## Data Availability

The original contributions presented in the study are included in the article/supplementary material, further inquiries can be directed to the corresponding author.

## Footnotes

^1^ https://www.credamo.com/

## References

[1] Luan J Filieri R Xiao J Han Q Zhu B Wang T. Product information and green consumption: an integrated perspective of regulatory focus, self-construal, and temporal distance. Inf Manag. (2023) 60:103746. doi: 10.1016/j.im.2022.103746

[2] Long F Jerath K Sarvary M. Designing an online retail marketplace: leveraging information from sponsored advertising. Mark Sci. (2022) 1:41. doi: 10.1287/mksc.2021.1307

[3] Sun F Deng X Jiao Y Li J Li J Xing H. Biochar-based fertilizer enhances carbon neutrality and net ecosystem economic benefit in tobacco (*Nicotiana tabacum* L) production. J Environ Manag. (2025) 387:125862. doi: 10.1016/j.jenvman.2025.125862, PMID: 40393118

[4] Chen LA House L. Food lifestyle patterns among contemporary food shoppers. Int J Consum Stud. (2022) 46:944–63. doi: 10.1111/ijcs.12739

[5] Han MS Hampson DP Wang Y Wang H. Consumer confidence and green purchase intention: an application of the stimulus-organism-response model. J Retail Consum Serv. (2022) 68:103061. doi: 10.1016/j.jretconser.2022.103061

[6] Guenther P Guenther M Ringle CM Zaefarian G Cartwright S. Improving PLS-SEM use for business marketing research. Ind Mark Manage. (2023) 111:127–42. doi: 10.1016/j.indmarman.2023.03.010

[7] Ashraf A Hameed I Saeed SA. How do social media influencers inspire consumers' purchase decisions? The mediating role of parasocial relationships. Int J Consum Stud. (2023) 47:1416–33. doi: 10.1111/ijcs.12917

[8] Gulfraz MB Sufyan M Mustak M Salminen J Srivastava DK. Understanding the impact of online customers’ shopping experience on online impulsive buying: a study on two leading E-commerce platforms. J Retail Consum Serv. (2022) 68:103000. doi: 10.1016/j.jretconser.2022.103000

[9] Goraya MAS Zhu J Akram MS Shareef MA Malik A Bhatti ZA. The impact of channel integration on consumers’ channel preferences: do showrooming and webrooming behaviors matter? J Retail Consum Serv. (2022) 65:102130. doi: 10.1016/j.jretconser.2020.102130

[10] He X Zhu L Sun L Yang L. The influence of brand marketing on consumers’ emotion in mobile social media environment. Front Psychol. (2022) 13:962224. doi: 10.3389/fpsyg.2022.962224, PMID: 35959055 PMC9360773

[11] Chen H Chen H Tian X. The dual-process model of product information and habit in influencing consumers’ purchase intention: the role of live streaming features. Electron Commerce Res Appl. (2022) 53:101150. doi: 10.1016/j.elerap.2022.101150

[12] Kaur J Mogaji E Paliwal M Jha S Agarwal S Mogaji SA. Consumer behavior in the metaverse. J Consum Behav. (2024) 23:1720–38. doi: 10.1002/cb.2298

[13] Benucci I Lombardelli C Esti M. A comprehensive review on natural sweeteners: impact on sensory properties, food structure, and new frontiers for their application. Crit Rev Food Sci Nutr. (2024) 17:1–19. doi: 10.1080/10408398.2024.2393204, PMID: 39154209

[14] Rahman SU Nguyen-Viet B. Towards sustainable development: coupling green marketing strategies and consumer perceptions in addressing greenwashing. Bus Strateg Environ. (2023) 32:2420–33. doi: 10.1002/bse.3256

[15] Hosseini Z Caneva G. Lost gardens: from knowledge to revitalization and cultural valorization of natural elements. Sustain For. (2022) 14:2956. doi: 10.3390/su14052956

[16] Huang Z Zhu Y Hao A Deng J. How social presence influences consumer purchase intention in live video commerce: the mediating role of immersive experience and the moderating role of positive emotions. J Res Interact Mark. (2023) 17:493–509. doi: 10.1108/JRIM-01-2022-0009

[17] Kouamé S Hafsi T Oliver D Langley A. Creating and sustaining stakeholder emotional resonance with organizational identity in social mission-driven organizations. Acad Manag J. (2022) 65:1864–93. doi: 10.5465/amj.2018.1143

[18] Júnior JR d O Limongi R Lim WM Eastman JK Kumar S. A story to sell: the influence of storytelling on consumers' purchasing behavior. Psychol Mark. (2023) 40:239–61. doi: 10.1002/mar.21758

[19] Chandra S Verma S Lim WM Kumar S Donthu N. Personalization in personalized marketing: trends and ways forward. Psychol Market. (2022) 39:1529–62. doi: 10.1002/mar.21670

[20] Mosikyan S Dolan R Corsi AM Bastian S. A systematic literature review and future research agenda to study consumer acceptance of novel foods and beverages. Appetite. (2024) 203:655. doi: 10.1016/j.appet.2024.107655, PMID: 39241833

[21] Chatzopoulou E. Sustainable brand resilience: mitigating panic buying through brand value and food waste attitudes amid social media misinformation. Sustain For. (2024) 16:658. doi: 10.3390/su16156658

[22] Ma Z Guo G Jing L. Consumer behavior research in the 21st century: clusters, themes, and future research agenda. Int J Consum Stud. (2024) 48:2980. doi: 10.1111/ijcs.12980

[23] Thakur M Kasi IK Islary P Bhatti SK. Nutritional and health-promoting effects of lichens used in food applications. Curr Nutr Rep. (2023) 12:6. doi: 10.1007/s13668-023-00489-6, PMID: 37581862

[24] Spence JT Helmreich R Stapp J. Personal attributes questionnaire. Dev Psychol. (1974)

[25] Connelly BL Certo TS Ireland DR Reutzel R Christopher R. Signaling theory: a review and assessment. J Manage. (2011) 37:39–67. doi: 10.1177/0149206310388419

[26] Kirmani A Rao AR. No pain, no gain: a critical review of the literature on signaling unobservable product quality. J Mark. (2000) 64:66–79. doi: 10.1509/jmkg.64.2.66.18000

[27] Ye Y Yang Y Huang Q. Identifying and examining the role of pop-up store design: a mixed-methods study. J Retail Consum Serv. (2023) 75:103503. doi: 10.1016/j.jretconser.2023.103503

[28] Mcgrew L Babos MB. Using a "talk show" presentation style to improve student engagement in virtual lectures. FASEB J. (2021) 35:141. doi: 10.1096/fasebj.2021.35.S1.04141

[29] Habibollahi F Kagan BJ Burkitt AN French C. Critical dynamics arise during structured information presentation within embodied in vitro neuronal networks. Nat Commun. (2023) 14:5287. doi: 10.1038/s41467-023-41020-3, PMID: 37648737 PMC10469171

[30] Sean M Kate F Sue C Lisa P Sarah Y Anuradhi J. Emotion processing and its relationship to social functioning and symptoms in psychotic disorder: a systematic review and meta-analysis. Schizophr Bull. (2024) 4:sbae167. doi: 10.1093/schbul/sbae167 PMC12236351 39360974

[31] Zilber TB. The work of the symbolic in institutional processes: translations of rational myths in Israeli high tech. Acad Manag J. (2006) 49:281–303. doi: 10.5465/amj.2006.20786073

[32] Abramo G D’Angelo CA Grilli L. The role of non-scientific factors Vis-à-Vis the quality of publications in determining their scholarly impact. Scientometrics. (2024) 129:5003–19. doi: 10.1007/s11192-024-05106-z

[33] Kim M Moon H Joo Y Yoon Y. Tourists' perceived value and behavioral intentions based on the choice attributes of wellness tourism. Int J Tourism Res. (2024) 26:2623. doi: 10.1002/jtr.2623

[34] Li X Wang Q Yao X Yan X Li R. How do influencers' impression management tactics affect purchase intention in live commerce? – trust transfer and gender differences. Inf Manag. (2025) 62:4094. doi: 10.1016/j.im.2024.104094

[35] Carneiro AMD Pacheco-Barrios K Andrade MF Martinez-Magallanes D Pichardo E Caumo W. Psychological factors modulate quantitative sensory testing measures in fibromyalgia patients: a systematic review and meta-regression analysis. Psychosom Med. (2024) 86:9. doi: 10.1097/PSY.0000000000001343 39225326

[36] Velasco Vizcaíno F Pohlmann A. Unpalatable solutions: consumer resistance towards insect-based foods is moderated by uncertainty avoidance. Psychol Mark. (2025) 42:142. doi: 10.1002/mar.22142

[37] Duplaga M Smola P Wojcieszko M. The interplay of technology anxiety and digital health literacy in determining e-health readiness. Eur J Pub Health. (2024) 34:1180. doi: 10.1093/eurpub/ckae144.1180

[38] Ren Y Wang X Peng W Yang K Kong X Jiang J. Investigation on the changes of perioperative psychological state of young patients with early breast Cancer. Psycho-Oncology. (2024) 33:e70027. doi: 10.1002/pon.70027, PMID: 39663427

[39] Balabanis G Stathopoulou A Balabanis J. Cultural influences on privacy calculus in loyalty programs: an analysis of individual and national-level cultural values. J Int Mark. (2025) 33:1069031X241262728. doi: 10.1177/1069031X241262728

[40] Jung JM Kellaris JJ. Cross-national differences in proneness to scarcity effects: the moderating roles of familiarity, uncertainty avoidance, and need for cognitive closure. Psychol Mark. (2004) 21:739–53. doi: 10.1002/mar.20027

[41] Yeung RM Morris J. An empirical study of the impact of consumer perceived risk on purchase likelihood: a modelling approach. Int J Consum Stud. (2006) 30:294–305. doi: 10.1111/j.1470-6431.2006.00493.x

[42] Mazzoni D Amadori R Sebri V Tosi M Pregnolato S Surico D. Health anxiety and oppressive support: their impact on decisions for non-urgent use of the emergency department of obstetrics and gynecology. Curr Psychol. (2024) 43:5. doi: 10.1007/s12144-023-05198-5

[43] Jiang S Zhou C. Stability and related zero viscosity limit of steady plane Poiseuille-Couette flows with no-slip boundary condition. J Differ Equ. (2025) 420:52–98. doi: 10.1016/j.jde.2024.11.047

[44] Moisan L Fournier PL Lavender M. Levers of social services integration: performance management system and lean-related management tools. Public Money Manag. (2023) 43:463–72. doi: 10.1080/09540962.2021.1996074

[45] Yang D Gui G Yao Y Ke X. Effect on consumers' sustainable purchase intention of dietary supplement purine labelling. Front Nutr. (2025) 12:1526713. doi: 10.3389/fnut.2025.1526713, PMID: 40535036 PMC12173902

[46] Tan S Xu X Shi H Song B. Joint alignment network preserving structural information for multimode process fault diagnosis. Can J Chem Eng. (2024) 102:106. doi: 10.1002/cjce.25106

[47] Wang Z. Digital mechanical Metasurfaces for reprogrammable structural display. ACS Nano. (2023) 17:10078–89. doi: 10.1021/acsnano.2c12679, PMID: 37260374

[48] Melo GFD Ornellas FR. A theoretical contribution to the characterization of the electronic structure and spectroscopic properties of the low-lying states of strontium monoiodide, SrI. J Quant Spectrosc Radiative Transfer. (2021) 268:107648. doi: 10.1016/j.jqsrt.2021.107648

[49] Ambruleviius F Valinius G. Electrochemical impedance spectrum reveals structural details of distribution of pores and defects in supported phospholipid bilayers. Bioelectrochemistry. (2022) 146:108092. doi: 10.1016/j.bioelechem.2022.108092 35367931

[50] Zhang Y Liu J Xiaoyong M. Value delivery in green consumption: the eect of advertisement value proposition on consumer perception and purchase intention. Front Psychol. (2024) 15:1339197. doi: 10.3389/fpsyg.2024.1339197 38323163 PMC10844521

[51] Galvin BM Badura K Lepine J Lepine M. A theoretical integration of leader emergence and leadership effectiveness: over, under, and congruent emergence. J Organiz Behav. (2024) 45:295. doi: 10.1002/job.2724

[52] Marty AP Braun J Schick C Zalunardo MP Spahn DR Breckwoldt J. A mobile application to facilitate implementation of programmatic assessment in anaesthesia training. Br J Anaesth. (2022) 6:128. doi: 10.1016/j.bja.2022.02.038 35410792

[53] Giuppone CA Gramajo LV Emmanuel G N RM Nicolás C Hinse TC. Unveiling hidden companions in post-common-envelope binaries: a robust strategy and uncertainty exploration. Astron Astrophys. (2024) 683:7030. doi: 10.1051/0004-6361/202347030

[54] Ji J Liu S Wang H Liu Y Jin M Sheng J. A boundary condition decoupled steady-state equivalent model for the simulation of FAIMS ion optical path. Int J Mass Spectrom. (2025) 508:7387. doi: 10.1016/j.ijms.2024.117387

[55] Varela OE Burke M Jáuregui K Quevedo S. External validity of teamwork and leadership behavior in academic labs: evidence from samples in Peru and the U.S. J Soc Psychol. (2022) 163:655–75. doi: 10.1080/00224545.2022.2144707 36416232

[56] Warneke K Orae M Plschberger G Herbsleb M Afonso J Wallot S. When testing becomes learning—underscoring the relevance of habituation to improve internal validity of common neurocognitive tests. Eur J Neurosci. (2025) 61:117. doi: 10.1111/ejn.70117 PMC12022537 40275720

[57] Chen H Zhou Y Zhang M Gong G Yue G Luo L. Molluscicidal effect mechanism study on metaldehyde to *Pomacea canaliculata* at low temperature. Pest Manag Sci. (2024) 80:15. doi: 10.1002/ps.8069 38456499

[58] Bennett CT. Untested admissions: examining changes in application behaviors and student demographics under test-optional policies. Am Educ Res J. (2022) 59:180. doi: 10.3102/00028312211003526

[59] Toldos-Romero MDLP Orozco-Gomez MM. Brand personality and purchase intention. Eur Bus Rev. (2015) 27:462–76. doi: 10.1108/EBR-03-2013-0046

[60] Kumpulainen T Vainio A Sandell M Hopia A. The effect of gender, age and product type on the origin induced food product experience among young consumers in Finland. Appetite. (2018) 123:101–7. doi: 10.1016/j.appet.2017.12.011, PMID: 29253668

[61] Wang Z Wang Z Rinprasertmeechai D Worawan S. Understanding the effects of live streamers’ appearance and abilities in shaping consumer purchase: a cross-cultural empirical research. J Retail Consum Serv. (2024) 81:104031. doi: 10.1016/j.jretconser.2024.104031

[62] Dash G Alharthi M Albarrak M Aggarwal S. Saudi millennials’ panic buying behavior during pandemic and post-pandemic: role of social media addiction and religious values and commitment. J Retail Consum Serv. (2024) 79:103891. doi: 10.1016/j.jretconser.2024.103891

[63] Meuter ML Bitner MJ Ostrom AL Brown SW. Choosing among alternative service delivery modes: an investigation of customer trial of self-service technologies. J Mark. (2005) 69:61–83. doi: 10.1509/jmkg.69.2.61.60759

[64] Shaouf A Lü K Li X. The effect of web advertising visual design on online purchase intention: an examination across gender. Comput Human Behav. (2016) 60:622–34. doi: 10.1016/j.chb.2016.02.090

[65] Song J Brown JM. The influence of "advancing" and "receding" colors on figure-ground perception under monocular and binocular viewing. Atten Percept Psychophys. (2024) 86:2707–20. doi: 10.3758/s13414-024-02956-w, PMID: 39349921 PMC11652407

[66] Wu Q Gorshkov M Pasa-Tolic L. Towards increasing the performance of FTICR-MS with signal detection at frequency multiples: signal theory and numerical study. Int J Mass Spectrom. (2021) 469:6669. doi: 10.1016/j.ijms.2021.116669

[67] Rahman RU Heinberg M Banerjee S Katsikeas CS. A good signal: how firms can utilize country of origin as a strategic analytical tool. J Int Mark. (2024) 32:4038. doi: 10.1177/1069031X241254038

[68] Ambika A Shin H Jain V. Immersive technologies and consumer behavior: a systematic review of two decades of research. Aust J Manage. (2025) 50:429. doi: 10.1177/03128962231181429

[69] Shao P Huang J Wang S Li Z. The emotional resonance and value recognition of digital memory: a short video communication study on the memory of a Chinese hero. Curr Psychol. (2024) 43:13654–67. doi: 10.1007/s12144-023-05410-6, PMID: 40589851

[70] Cawood A Smith C Kinnear F Upton L Trace S O'Connor G. Effect of oral nutritional supplements on outcomes in children presenting with, or at risk of, faltering growth in clinical settings: a systematic review and meta-analysis. J Child Health Care. (2025) 29:181. doi: 10.1177/13674935231185181, PMID: 37406354

[71] Hoffman S Signorini G Soldavini AM Simons CT. Implementing a discrete choice experiment within consumer sensory evaluation to better understand purchase intention. J Sens Stud. (2025) 40:70006. doi: 10.1111/joss.70006

[72] Zhu C Liu Z Pei M Su Y. Effects of photographer physical attractiveness on photograph aesthetic value assessment. Perception. (2022) 51:505–13. doi: 10.1177/03010066221098158, PMID: 35581900

[73] Septianto F Ye S Northey G. The effectiveness of advertising images in promoting experiential offerings: an emotional response approach. J Bus Res. (2021) 122:15. doi: 10.1016/j.jbusres.2020.09.015

[74] Gradojevic N Kukolj D. Unlocking the black box: non-parametric option pricing before and during COVID-19. Ann Oper Res. (2024) 334:59–82. doi: 10.1007/s10479-022-04578-7, PMID: 35233127 PMC8874738

[75] Chen X Wang Y Wei S Shen J. The effect of herd behavior on consumer intention in live streaming e-commerce: the moderating role of interaction. Int J Hum Comput Interact. (2025) 41:1674–87. doi: 10.1080/10447318.2024.2364464

[76] Cuadros-Rodríguez L Arroyo-Cerezo A Jiménez-Carvelo AM. Revamping information entropy: a tailored metric for pre-evaluating quality of 2D analytical signal – a tutorial. Anal Chim Acta. (2025) 1344:343693. doi: 10.1016/j.aca.2025.343693, PMID: 39984219

[77] Bao Y Li F Chen L Mu Q Wen D. Fate of antibiotics in engineered wastewater systems and receiving water environment: a case study on the coast of Hangzhou Bay, China. Sci Total Environ. (2021) 769:144642. doi: 10.1016/j.scitotenv.2020.144642 33736269

[78] Park SY. Editorial comment: toward routine application of dual-energy CT for genitourinary assessment—a perspective in the emergency setting. Am J Roentgenol. (2023) 221:731. doi: 10.2214/AJR.23.29510, PMID: 37098968

[79] Kondo H Nakamura Y. Light emission phenomenon and glare in visual environment design. J Environ Engineer. (2023) 88:22–9. doi: 10.3130/aije.88.22

[80] Sady S. Behavioral intention to purchase sustainable food: generation z's perspective. Sustain For. (2024) 16:7248. doi: 10.3390/su16177284

[81] Su L Lai Z Huang Y. How do tourism souvenir purchasing channels impact tourists' intention to purchase? The moderating role of souvenir authenticity. J Travel Res. (2024) 63:1527–48. doi: 10.1177/00472875231195062, PMID: 40575798

[82] You L Liu F. From virtual voices to real impact: authenticity, altruism, and egoism in social advocacy by human and virtual influencers. Technol Forecast Soc Change. (2024) 207:650. doi: 10.1016/j.techfore.2024.123650

[83] Fawns T Schuwirth L. Rethinking the value proposition of assessment at a time of rapid development in generative artificial intelligence. Med Educ. (2024) 58:14–6. doi: 10.1111/medu.15259, PMID: 37882469

[84] Artykbayeva F Spatay A Raimov A Bakirova S Taiteliyeva M. Value characteristics of the Core of the mental lexicon of native speakers of language and culture in the light of intercultural communication. J Psycholinguist Res. (2024) 53:32. doi: 10.1007/s10936-024-10074-9, PMID: 38526840

[85] Aung N Dhelim S Kechadi LCLZNCT. Vesonet: traffic-aware content caching for vehicular social networks using deep reinforcement learning. IEEE Trans Intell Transp Syst. (2023) 24:8638–49. doi: 10.1109/TITS.2023.3250320

[86] Dermody J Koenig-Lewis N Zhao AL Hanmer-Lloyd S. Critiquing a utopian idea of sustainable consumption: a post-capitalism perspective. J Macromark. (2021) 41:9148. doi: 10.1177/0276146720979148

[87] Tzeng SY Ho TY. Exploring the effects of product knowledge, trust, and distrust in the health belief model to predict attitude toward dietary supplements. SAGE Open. (2022) 12:58–72. doi: 10.1177/21582440211068855

[88] Domzaridou E Carr MJ Millar T Webb RT Ashcroft DM. Recognizing the complexities of co-prescriptions and life-style factors in opioid agonist treatment: a response from Eleni Domzaridou, Matthew J. Carr, Tim Millar, Roger T. Webb and Darren M. Ashcroft. Addiction. (2024) 119:2. doi: 10.1111/add.16447 38317288

[89] Margaret MC. Advertising or evidence?—why we need system changes in academia to improve media reporting. JAMA Intern Med. (2021) 181:242. doi: 10.1001/jamainternmed.2021.0242 33818594

[90] Reijnen E Laasner Vogt L Catarci D Zengaffinen JL Bremermann-Reiser SM Bluer L. Humor and the willingness to buy healthy food posted on Instagram. Front Psychol. (2024) 15:9648. doi: 10.3389/fpsyg.2024.1419648 PMC11347272 39193037

[91] Xu P Ye P Zhao F Jahanger A. Technology spillover and market competitiveness in green credit induced corporate green innovation: an evolutionary game theory and empirical study. Technol Forecast Soc Change. (2024) 207:622. doi: 10.1016/j.techfore.2024.123622

[92] Kang SY Kim J Lakshmanan A. Anatomical Depiction: How Showing a Product’s Inner Structure Shapes Product Valuations. J Market. (2025) 89:56–76. doi: 10.1177/00222429241257911

